# A Remedy for Neuralgia without Morphine

**Published:** 1890-05

**Authors:** 


### A REMEDY FOR NEURALGIA WITHOUT MORPHINE.

| | | |
|---|---|---|
| ℞. | Antipyrin - - - - - | ʒiij. |
| | Ex. cannabis Ind. | |
| | Ex. aconite - - - - - | aa gr. vss. |
| | Caffein - - - - - | ʒss. |
| | Hyoscine hydrobrom - - - | gr. 1-3. |

Divide into thirty capsules.

—*Journal of American Medical Association.*

The following cough mixture is highly successful and does not disorder the stomach:

| | | |
|---|---|---|
| ℞. | Morphin. bimeconatis - - - - | gr. j. |
| | Ammon. muriatis - - - - - | ℥j. |
| | Aquæ camphoræ - - - - - | ℥iss. |
| | Aquæ q. s. ad. - - - - - | ℥iij. |

Sig. One teaspoonful as required.

—*Journal of the Respiratory Organs.*

